# Otologic and audiologic findings in 22q11.2 deletion syndrome

**DOI:** 10.1007/s00405-016-4365-y

**Published:** 2016-11-11

**Authors:** E. Verheij, A. L. Kist, A. B. Mink van der Molen, I. Stegeman, G. A. van Zanten, W. Grolman, H. G. X. M. Thomeer

**Affiliations:** 1 Department of Otorhinolaryngology-Head and Neck Surgery, University Medical Center Utrecht, Heidelberglaan 100, 3584 CX Utrecht, The Netherlands; 2 Brain Center Rudolf Magnus, University Medical Center Utrecht, Utrecht, The Netherlands; 3 Department of Plastic Surgery, University Medical Center Utrecht, Utrecht, The Netherlands

**Keywords:** DiGeorge syndrome, Velocardiofacial syndrome, 22q11.2 deletion syndrome, Hearing loss, Otitis media, Tympanic membrane perforation, Otorhinolaryngology

## Abstract

Hearing loss is frequently present in the 22q11.2 deletion syndrome. Our aim was to describe the audiologic and otologic features of patients with 22q11.2 deletion syndrome. We conducted a retrospective cohort study in a single tertiary referral center. We reviewed medical files of all patients with 22q11.2 deletion syndrome who visited an otolaryngologist, plastic surgeon or speech therapist, for audiologic or otologic features. Hearing loss was defined as a pure tone average (of 0.5, 1, 2, and 4 kHz) of >20 decibel hearing level. Audiograms were available for 102 of 199 included patients, out of which 163 ears were measured in the required frquencies (0.5–4 kHz). Median age at time of most recent audiogram was 7 years (range 3–29 years). In 62 out of 163 ears (38%), hearing loss was present. Most ears had conductive hearing loss (*n* = 58) and 4 ears had mixed hearing loss. The severity of hearing loss was most frequently mild (pure tone average of ≤40 decibel hearing level). In 22.5% of ears, otitis media with effusion was observed at time of most recent audiogram. Age was not related to mean air conduction hearing thresholds or to otitis media with effusion (*p* = 0.43 and *p* = 0.11, respectively). In conclusion, hearing loss and otitis media are frequently present in patients with 22q11.2 deletion syndrome. Moreover, our results suggest that children with 22q11.2 deletion syndrome remain susceptible for otitis media as they age.

## Introduction

First identified in 1983 and later confirmed in the 1990s, patients with velocardiofacial syndrome, DiGeorge syndrome, and conotruncal anomaly face syndrome were found to have a microdeletion in the same genetic region, the 22q11.2 region [1–4]. This led to one united syndrome, the 22q11.2 deletion syndrome (22q11DS) [5]. This syndrome has a heterogenic phenotype and is characterized by congenital heart anomalies, immunodeficiency, kidney abnormalities, cleft palate (from bifid uvula to complete cheilo-gnatho-palato cleft), velopharyngeal insufficiency, speech, and language impairment [5–10]. Many patients diagnosed with this syndrome are known to have recurrent otitis media and hearing loss [5–18]. The reported prevalence of hearing loss in 22q11DS varies between 40 and 64.5%, which is considerably higher compared to the prevalence rate in the general population [11–19]. Hearing loss in 22q11DS is mostly conductive, but sensorineural and mixed hearing loss is also described [11–18]. Conductive hearing loss in patients diagnosed with 22q11DS is associated with recurrent otitis media [10–12, 14, 16–18]. Causes of otitis media and conductive hearing loss in 22q11DS are presumably multifactorial. Many patients with 22q11DS suffer from immunodeficiency with recurrent respiratory tract infections. In addition, dysfunction of the Eustachian tube is suggested to be an important factor in developing otitis media [11, 12, 14, 17]. Mouse models of 22q11DS have shown a relation between otitis media and conductive hearing loss [20, 21]. In addition, in mouse models of 22q11DS, a hypoplastic levator veli palatini muscle, an intrinsic muscle of the Eustachian tube, was found. Interestingly, in the case of unilateral otitis media with effusion, the levator veli palatini muscle in mice was significantly smaller on the side of otitis media compared to the non-inflamed side [22]. In addition, congenital middle ear malformations are also described in patients [18, 23, 24].

Regarding the sensorineural hearing loss, cochlear damage as a result of chronic otitis media has been suggested as a possible underlying cause [15, 17]. Furthermore, *Tbx1*, a gene located on the 22q11.2 region (the same region where the microdeletion in 22q11DS is located), is suggested to be required for inner ear development [25, 26]. Along with this finding, congenital malformation of the cochlea is described in a case report [23].

Our tertiary referral center contains a cohort of approximately 220 22q11DS patients who are evaluated and treated by a multidisciplinary team. We aimed to describe the otologic and audiologic findings of these patients. In addition, we analyzed the influence of aging on hearing thresholds and otologic manifestations.

## Materials and methods

### Patients

We conducted a retrospective cohort study in a single Dutch tertiary referral center. All patients diagnosed with 22q11DS after multidisciplinary outpatient intake and examination (including plastic surgeon, otolaryngologist, and speech therapist) until 12 November 2015 were included. We reviewed medical files for audiologic and otologic features including a history of otitis media [acute or chronic (with effusion)], grommet insertion, tympanic membrane perforation, cholesteatoma, adenoidectomy, adaptation of hearing aids (air or bone conducted) and history of otologic surgery. In addition, we collected all available conventional pure tone audiograms and we reviewed otoscopic reports specifically at time of the most recent audiogram. If these reports were lacking, information on tympanic membrane perforations and grommets could be reasoned if there was a tympanic membrane perforation or grommet in place before and after the most recent audiogram. Then we assumed that those findings were also present during the most recent audiogram.

### Audiometric examination

We defined hearing loss as a pure tone average (PTA) (at 0.5, 1, 2 and 4 kHz) of more than 20 decibel hearing level (dB HL), in concordance with the AAO-HNS 1995 guidelines (apart from 3 kHz where we used 4 kHz) [27]. Conductive hearing loss was determined as an average air conduction (AC) threshold of >20 dB HL, and the air bone gap (ABG) was ≥10 dB at one or more frequencies. Sensorineural hearing loss was defined as hearing loss with an ABG <10 dB in all frequencies and mixed hearing loss as an average AC and bone conduction (BC) threshold of >20 dB HL, and an ABG of ≥10 dB at one or more frequencies. Hearing loss was classified as mild (21–40 dB), moderate (41–60 dB), moderate to severe (61–70 dB), severe (71–90 dB), and profound (≥91 dB). In the cases of absent bone conduction measurement at first or the most recent audiometric evaluation, BC thresholds from earlier or later measurements were evaluated. If previous or later BC measurements were lacking, the BC from the contralateral ear was adapted. We defined immeasurable AC thresholds due to bad hearing (marked by a downward arrow on the audiogram) as a threshold of 130 dB HL. For immeasurable BC thresholds with measurable AC, consensus between authors (EV, BvZ, and HT) was reached on how to interpret these findings. When two audiograms or more were performed with an interval of at least 1 year, we compared the hearing thresholds from the first and the most recent PTA.

### Statistical processing and analysis

Due to various practical reasons, in some audiograms, not all hearing thresholds were measured for every frequency. Those missing data were assumed to be missing at random, implying that the missing at random (MAR) assumption was applicable. Therefore, multiple imputation was used to handle missing hearing thresholds [28]. We generated ten imputation sets.

We used the Mann–Whitney *U* test to analyze the relation between age and otologic pathology. Linear regression was used to evaluate the effect of age on hearing thresholds. In this evaluation, we did one sensitivity analysis where we excluded outliers. We employed SPSS version 21 for statistical analysis.

## Results

### Missing data

BC hearing thresholds were not measured in 79 ears in the most recent audiogram. Earlier measurements were evaluated in 28 ears, and the BC thresholds from the contralateral ear were adapted in 41 ears. In five patients and ten ears, the BC was never measured, nor was there a BC threshold measured of the contralateral ear. All these ears had AC conduction thresholds in the normal range. In these cases, since they had normal hearing, we assumed that there was no ABG. In 49 ears, where there were at least two audiograms with an interval of at least 1 year, BC thresholds were not measured in the first audiogram. Later measurements were evaluated in 38 ears, and the BC thresholds that form the contralateral ear were adapted in 11 ears. Next, in the most recent and first pure tone audiogram, there were 16% and 25.5% missing hearing thresholds, respectively.

### Medical history

We included a total of 199 patients, 102 males, and 97 females. Audiometric measurements were available for 102 patients and 204 ears. Characteristics are shown in Table 1. Median age at most recent audiogram was 7 years (range 3–29 years). Median age at start of study was 11 years (range 2.5 months–30 years). Many patients had an otologic history; in 61% of patients, grommets were inserted, varying between once to 17 times. 15 patients were adapted with conventional hearing aids and four patients with a bone conduction device (BCD) on a softband. One patient received a percutaneous BCD, but was a non-user. Six patients were using an assistive listening device at school, coupled to a conventional hearing aid in four patients and to ear phones in two patients. 14 patients underwent otologic surgery, comprising more than one surgical intervention in six of these patients. One patient received a canalplasty for an acquired stenosis of the external auditory canal. Five out of 14 patients underwent middle ear surgery for chronic otitis media, including mastoidectomy, attico-antrotomy or a combination of surgical approaches to treat the pathology. Ten out of 14 patients underwent tympanoplasty, two patients in both ears, and seven patients in one ear. In four out of 14 patients, an epithelial rim boarding the tympanic membrane perforation was removed to stimulate spontaneous closure. In addition, 47 patients (25%) underwent adenoidectomy.

**Table 1:** Demographic characteristics and medical history of 199 patients

	Patients (*n* = 199) (%)
Male	102 (51)
Audiogram available	102 (51)
Median age in years at most recent audiogram (range) (*n* = 102 patients)	7 (3–29)
History of ventilation tubes (*n* = 186 patients)	113 (61)
History of adenoidectomy (*n* = 192 patients)	47 (24)
History of cholesteatoma (*n* = 192 patients)	0 (0)
History of tympanic membrane perforation (*n* = 190 patients)	55 (29)
Use of hearing aids/BCD (*n* = 194 patients)	19 (10)
Otologic surgery (*n* = 194 patients)	14 (7)

*PTA* pure tone average, *BCD* bone conduction device

Considering otoscopic reports at time of the most recent audiograms, a tympanic membrane perforation was present in 31 of 196 ears (15.8%). Grommets were present and patent in 29 of 185 ears (15.7%), otitis media with effusion (OME) was present in 32 of 142 ears (22.5%), and 22 of these 32 ears had a history of grommet insertion. In addition, 2 of 142 ears (1.4%) had acute otitis media (OMA) while a grommet in place (purulent otorrhoea).

### Most recent audiogram

There were two ears with immeasurable BC thresholds with measurable AC thresholds. In one of those two ears, there was an immeasurable BC threshold at 4 kHz, where the AC threshold at 4 kHz was 100 dB HL, the frequencies 0.5–2 kHz showed an ABG of 10–30 dB, and we interpreted this audiogram as mixed hearing loss. The other ear had immeasurable BC thresholds at 0.5 and 1 kHz with AC thresholds of 60 and 65 dB HL, interpreted as a pure sensorineural hearing loss in the low frequencies, but overall as a mixed hearing loss, because the ABG was 20 dB at 2 and 4 kHz.

**Fig. 1:**
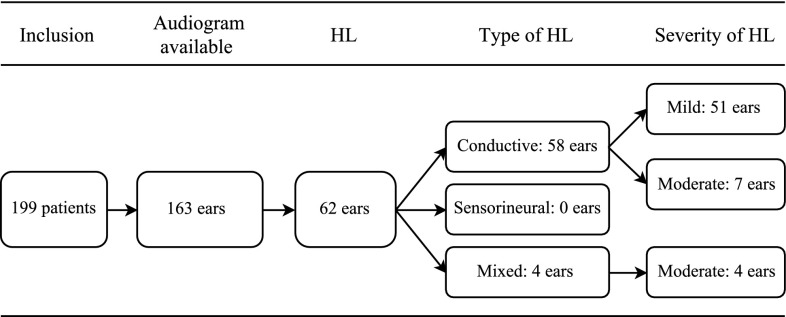
Flowchart of audiogram results in 163 patients. *HL* hearing loss

Overall, patients had received their most recent audiogram between 1995 and 2015. The median PTA AC threshold was 17.5 dB HL (range −1.3 to 57.5 dB HL), and the median of PTA BC threshold was 2.4 dB HL (range −9.4 to 46.3 dB HL). There were 163 ears where every frequency (0.5, 1, 2, and 4 kHz) was measured and hearing loss was found in 62 of these ears (38%) (Fig. 1). After imputation of missing data, there were 77 of 204 ears with hearing loss (38%). Most of which suffered from pure conductive hearing loss.

Frequently, hearing loss was mild, but seven ears belonging to seven patients had a moderate conductive hearing loss. Two of these ears were diagnosed with OME at the time of audiometric evaluation, one ear had a tympanic tube in place and otitis, one ear had a tympanic membrane perforation, one ear had no any otologic problem at time of audiometric evaluation, and finally, two ears had no available otoscopic information. One of these two ears had an acquired stenosis of the external auditory canal after chronic obliterative otitis externa. The other ear without an obvious cause for the hearing loss had tympanosclerosis involving the ossicular chain, shown on a computed tomography (CT) scan (patient’s history revealed chronic otitis media).

In four ears (three patients), a sensorineural component in combination with a conductive hearing loss (mixed hearing loss) was observed. In all of these ears, the severity of hearing loss was moderate with a median of PTA AC threshold of 50.6 dB HL (range 46.3–56.3 dB HL) and a median of PTA BC threshold of 37.5 dB HL (range 33.8–46.3 dB HL). CT scanning of one patient (out of three) with bilateral mixed hearing loss revealed soft tissue opacification of the right middle ear, but no malformations. Another patient with unilateral mixed hearing loss had an anterior inferior cerebellar artery loop on the same side (left) as the ear with mixed hearing loss, shown on magnetic resonance imaging of the petrous bone including the cerebellopontine angle. However, no specific cause for the hearing loss could be found. The last patient with unilateral mixed hearing loss was diagnosed with a tympanic membrane perforation in both ears, but no other pathology potentially causing the mixed hearing loss was identified.

### Hearing thresholds in relation to age

**Fig. 2:**
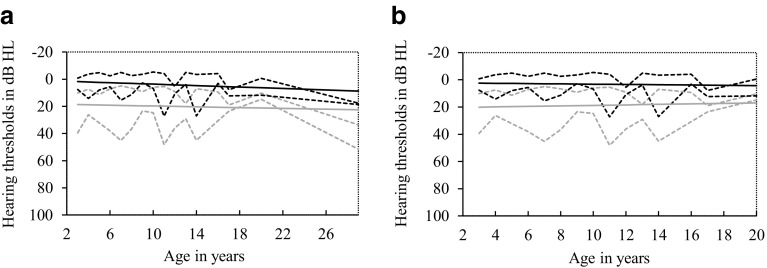
Average hearing thresholds plotted against age and linear regression lines. P5 and P95 of bone conduction (*black*) and air conduction (*gray*) thresholds are shown in *dotted lines*, *linear regression lines* are shown in *continuous lines*. **a** Linear regression analysis: no relation between age and air conduction thresholds (*p* = 0.43), and a significant relation between age and bone conduction thresholds (*p* = 0.03). **b** Results without two outliers. Linear regression analysis: no relation between age and air conduction thresholds or bone conduction thresholds (*p* = 0.38 and *p* = 0.46, respectively)

Figure 2 shows the mean AC and BC thresholds plotted against the age of patients. The linear regression line shows no relation between hearing thresholds and age for average AC thresholds (*p* = 0.43). However, age was significantly related to  the average BC thresholds (*p* = 0.03), and the slope for this linear regression line was very small (0.27). There were two outliers (two ears from one patient), aged 29 years with poor hearing. In the sensitivity analysis without these outliers, there was no significant relation between age and AC or BC hearing thresholds (*p* = 0.38 and *p* = 0.46 for average AC and BC hearing thresholds, respectively). The median age of patients with OME during the most recent audiogram was 7 years (range 3–14 years). Age at time of the most recent audiogram was not related to the presence of OME (*p* = 0.11).

### Progression over time

**Fig. 3:**
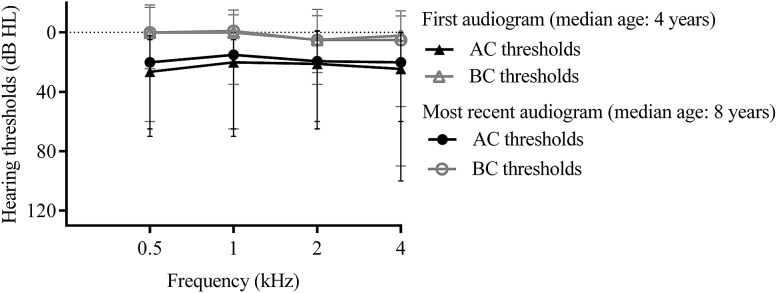
Median thresholds of the first and most recent audiograms of 104 ears

In 104 ears, at least two audiograms were available with an interval of at least 1 year (Fig. 3). 31 of these 104 ears showed worsening of the AC threshold at 1 kHz [median decrease 10 dB (range 5–50 dB)], 18 ears had exactly the same PTA over time, and 55 of 104 ears showed improvement [median improvement 10 dB (range 5–35 dB)].

In 23 of 104 ears, at least 20 dB difference was shown (range 20–50 dB) between the first and most recent AC thresholds at 1 kHz. Nine of these 23 ears showed worsening (median 20 dB), whereas the remaining 14 ears showed improvement (median 27 dB). Out of those 23 ears, 13 ears were diagnosed with an otologic abnormality during otoscopy at time of the first or most recent audiogram, four had a tympanic membrane perforation, six ears had (otoscopically confirmed) OME, and two ears had OMA in combination with a grommet in place. One ear had a recurrent stenosis of the external auditory canal, not present at time of the first audiogram.

**Fig. 4:**
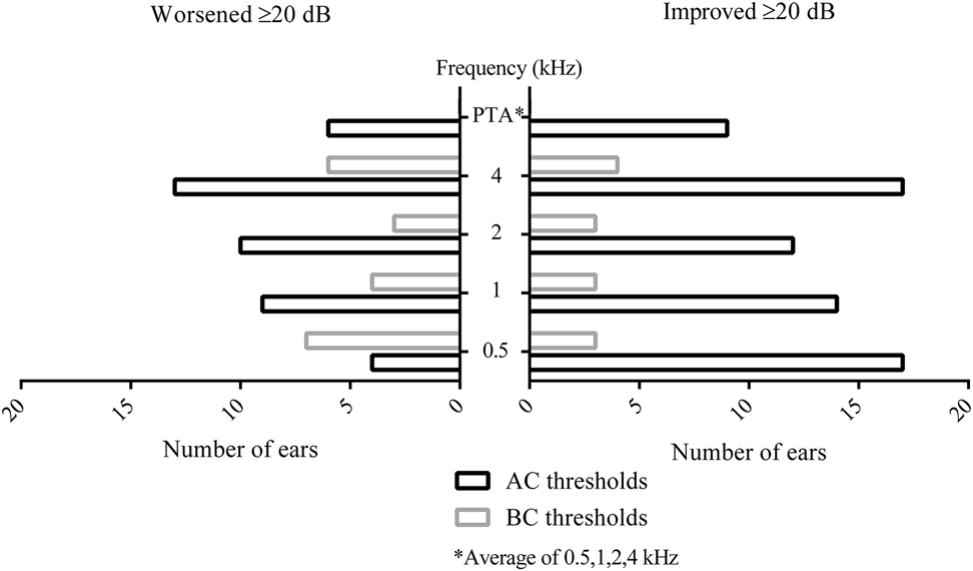
Numbers of ears with an improvement or worsening of ≥20 dB between the first and most recent audiograms. *PTA* pure tone average

For BC at 1 kHz, 37 of 104 ears had the same hearing level over time, 40 ears showed improvement [median improvement was 5 dB (range 4–20 dB)], and 27 ears (24 ears before multiple imputation) showed worsening [median decrease 9 dB (range 4–26 dB)]. Seven of 104 ears showed a difference of ≥20 dB in BC at 1 kHz between the first and most recent audiograms. Evaluation of other frequencies shows a roughly similar trend (Fig. 4).

## Discussion

We described the otologic features of 199 patients and the audiometric results of 163 ears in patients with 22q11DS. Hearing loss was frequently present in our study population and was predominantly conductive with a mild severity. Previous studies regarding 22q11DS report similar results [11, 12, 14, 16–18]. Conductive hearing loss in patients diagnosed with 22q11DS is associated with recurrent otitis media [10–12, 14, 16–18]. Consistent with this finding, the cause of hearing loss in the present population is also related to otitis media. This appeared in different forms: some children suffered from OME at time of audiometric evaluation, some were diagnosed with a tympanic membrane perforation, which resulted after otitis media or grommet insertion, while another patient showed damage in the middle ear possibly due to chronic otitis media (tympanosclerosis involving the ossicular chain).

At time of the most recent audiogram, 22.5% of all ears had OME. This number is higher compared to the normal population, where the reported prevalence of children aged 7.5–8 years is around 6% [29]. In another study regarding healthy children with a broad age range (5–14 years), such as our population, an overall prevalence of OME of 6.8% was found [30]. In (non-syndromatic) children with a cleft palate, otitis media is also very common and suggested to be caused by Eustachian tube dysfunction [31, 32] A cleft (mostly submucosal) is frequently present in the 22q11DS population [5, 10]. Possibly, in patients with a cleft and in patients with 22q11DS, otitis media is caused by the same pathophysiology. Especially, since in mouse models for 22q11DS, a hypoplastic levator veli palatini muscle was found [22].

AC hearing thresholds seemed not to change with aging in our population. Moreover, age was not related to OME during the most recent audiogram. This suggests that children with 22q11DS continue to be at risk for otitis media with effusion as they age. This is consistent with Reyes et al. who found the same prevalences of OME in age younger than 3, 4–7 years and older than 7 years [12]. In the normal population, the prevalence peak of OME is between 6 months and 4 year of age, after that age the prevalence decreases [33–35].

Surprisingly, none of our patients had a pure sensorineural hearing loss and mixed hearing loss was only seen in four ears. This prevalence is considerably lower compared to other studies [11–13, 15–18]. One possible explanation for our low rate of sensorineural hearing loss is the fact that pure tone audiograms were only available in 51% of our study population. Theoretically, patients with mild hearing loss could have been diagnosed more often in a general hospital rather than in our tertiary care center. This would underestimate our reported prevalence of hearing loss. However, this is not supported by the fact that most of our patients with an available audiogram had mild hearing loss. Furthermore, the rate of patients with hearing loss with a sensorineural component varies much in reported studies (2.8–19.4%) [11–13, 15–18]. Patient ages were different among these studies, whereby three studies included older patients (mean 15, 16, and 24 years) [13, 15, 17], compared to the other studies [11, 12, 14, 16, 18]. In two studies with young patients audiometric testing involved behavioral pure tone audiometry or sound field testing [11, 16]. Although it is not possible to obtain pure tone audiometry in young non-cooperative children, these tests are less accurate than the conventional pure tone audiometry. In addition, Zarchi et al. and Van Eynde et al. found sensorineural hearing loss more prominent in the high tones (Zarchi et al. tested frequencies 0.25–8 kHz and Van Eynde et al. frequencies 0.125–11.2 kHz) [15, 17]. Due to our retrospective design, we only used the frequencies 0.5–4 kHz. Possibly, we missed poor BC thresholds in the high tones, which might explain our lower sensorineural hearing loss prevalence.

### Limitations

The main limitation of this study was that it was performed in a tertiary referral center. Some of the more regular pathology such as otitis media or tympanic membrane perforation will presumably be treated in primary or secondary care. Our number of tympanic membrane perforations, a history of ventilation tubes or adenoidectomy could therefore be underestimated. Another limitation is the fact that audiograms were available in 51%; selection bias is likely since patients with assumed normal hearing are less likely to undergo audiometric evaluation. In addition, patients with OME or other otologic anomalies are more likely to receive an audiogram; therefore, our results for otologic manifestations at time of most recent audiogram are likely overestimated.

In conclusion, hearing loss and otitis media are frequently present in patients with 22q11DS. Moreover, our results suggest that children with 22q11DS remain susceptible for otitis media as they age. Although conductive hearing loss is presumably largely caused by otitis media, future studies are needed to assess the cause of sensorineural and conductive hearing loss.
